# Improving enteral nutrition tolerance and protein intake maybe beneficial to intensive care unit patients

**DOI:** 10.1038/s41598-023-49050-z

**Published:** 2023-12-07

**Authors:** Ming Zhong, Yuzhen Qiu, Tingting Pan, Ruilan Wang, Yuan Gao, Xuebin Wang, Yingchuan Li, Zhaofen Lin, Zhixiong Wu, Jianguo Tang, Xiang Li, Xuemin Wang, Jiayu Zhang, Gang Feng, Sheng Wang, Xinyuan Lu, Ye Gong, Hongping Qu, Erzhen Chen

**Affiliations:** 1 Department of Critical Care Medicine, Ruijin Hospital, Shanghai Jiao Tong University School of Medicine, Shanghai, China; 2 Department of Critical Care Medicine, Shanghai General Hospital, Shanghai Jiao Tong University School of Medicine, Shanghai, China; 3 Department of Emergency and Critical Care Medicine, Renji Hospital, Shanghai Jiao Tong University School of Medicine, Shanghai, China; 4 Department of Critical Care Medicine, Shanghai East Hospital, Tongji University School of Medicine, Shanghai, China; 5 Department of Emergency and Critical Care Medicine, The Sixth People’s Hospital of Shanghai, Shanghai Jiao Tong University School of Medicine, Shanghai, China; 6 Department of Emergency and Critical Care Medicine, Shanghai Changzhen Hospital, Shanghai, China; 7 Department of Emergency and Critical Care Medicine, Huadong Hospital, Shanghai, China; 8 Department of Emergency and Critical Care Medicine, The Fifth People’s Hospital of Shanghai, Shanghai, China; 9 Department of Emergency and Critical Care Medicine, Central Hospital of Minghang District, Shanghai, China; 10 Department of Emergency and Critical Care Medicine, Central Hospital of Songjiang District, Shanghai, China; 11 Department of Emergency and Critical Care Medicine, Central Hospital of Putuo District, Shanghai, China; 12 Department of Emergency and Critical Care Medicine, Gongli Hospital of Pudong New Area, Shanghai, China; 13 Department of Emergency and Critical Care Medicine, The Tenth People’s Hospital of Shanghai, Shanghai, China; 14 Department of Emergency and Critical Care Medicine, Shanghai Pudong New Area People’s Hospital, Shanghai, China; 15 Department of Critical Care Medicine, Huashan Hospital, Fudan University, Shanghai, China; 16 Department of Emergency, Ruijin Hospital, Shanghai Jiao Tong University School of Medicine, Shanghai, China; 17 Shanghai Quality Improving Center of Critical Care Medicine, Shanghai, China

**Keywords:** Diseases, Gastroenterology, Signs and symptoms

## Abstract

Enteral nutrition (EN) is important for critically ill patients. This study investigated the current situation of EN treatment in SHANGHAI intensive care units (ICUs). We hypothesized that improving EN practice in SHANGHAI may benefit the prognosis of ICU patients. Clinical information on EN use was collected using clinic information forms in 2019. The collected data included the patient’s general clinical information, EN prescription status, EN tolerance status, and clinical outcomes. The observation time points were days 1, 3, and 7 after starting EN. A total of 491 patients were included. The proportion of EN intolerance (defined as < 20 kcal/kg/day) decreased, with rates of intolerance of 100%, 82.07%, 70.61%, and 52.23% at 1, 3, 7, and 14 days, respectively. Age, mNutric score, and protein intake < 0.5 g/kg/day on day 7 were risk factors for 28-day mortality.The EN tolerance on day 7 and protein intake > 0.5 g/kg/day on day 3 or day 7 might affect the 28-day mortality. Risk factors with EN tolerance on day 7 by logistic regression showed that the AGI grade on day 1 was a major factor against EN tolerance. The proportion of EN tolerance in SHANGHAI ICU patients was low. Achieving tolerance on day 7 after the start of EN is a protective factor for 28-day survival. Improving EN tolerance and protein intake maybe beneficial for ICU patients.

## Introduction

Enteral nutrition (EN) is the preferred nutrition route for critically ill patients and is widely adopted in intensive care units (ICUs)^1^. Still, many problems remain regarding the safety and effective application of EN, among which EN intolerance is one of the most important. EN intolerance is associated with prolonged hospital stays and increased mortality^2–4^. The European Critical Care Association and the Asia Society for Emergency and Critical Care Medicine recommended that the caloric supply reached 20 kcal/kg/day within 72 h from the start of EN as an objective standard of EN intolerance^5, 6^. “If the patient can use EN safely, then the patient will be saved”^7^. Therefore, ensuring the effectiveness and safety of EN is an important issue in clinical practice^8^.

The underfeeding of EN in China has been reported. In 2017, Li et al^9^. carried out a national nutrition survey covering 116 ICUs with a total of 1900 patients and showed that 60% of patients met the target on the 5–7th day. This results strongly indicated a high rate of EN intolerance in the ICUs across mainland China.Meanwhile, It should be noted that there are significant differences in the medical resources among different regions in China, which may lead to different treatment preferences in ICU nutrition support. Therefore, we conducted this investigation on the implementation of EN in SHANGHAI, especially on the characteristics of EN intolerance. We hope it will be helpful for making further efforts on improving EN application.

## Materials and methods

### Study design

This observational study included consecutive patients from the ICUs of 15 hospitals in SHANGHAI admitted from January to December 2019. The clinical data were collected according to the designed case report form. This study was approved by the Ethics Committee of our Hospital. As the major purpose is to the relation between EN tolerance and survival in ICU, this is made in a perspective way.

### Study population

#### Inclusion criteria

- (1) Patients admitted to the ICU of 15 hospitals who consented and agreed to participate in this research.
- (2) Patients aged > 18 years.

#### Exclusion criteria

1. Patients aged > 90 years.
2. ICU stay < 72 h.
3. Patients with absolute contraindications to EN, such as uncontrolled high-flow gastrointestinal fistula, unresolved intestinal obstruction, etc.
4. Patients with uncontrolled malignancy or end-stage chronic organ failure (heart, lung, liver, kidney, etc.)

### Data collection

The patient’s information was recorded in a case information sheet that included:

1. Nutritional risk assessment: NRS2002 score and mNutric score on ICU admission.
2. Nutrition prescription (days 1, 3, 7, and 14 after ICU admission): the actual calorie of EN and parenteral nutrition (PN), the amount of protein and amino acid.
3. Data related to EN tolerance (days 1, 3, 7, and 14 after ICU admission): actual calorie and protein intake, AGI score, and gastrointestinal symptoms. EN tolerance was defined as the caloric supply of the patient reaching 20 kcal/kg/day within 72 h from the start of EN^5, 6^. The symptoms of FI defined according to the judgement of ICU physician or ESICM Working Group on Abdominal Problems^5^.
4. Patients’ outcomes: 28-day mortality and in-ICU mortality.

As for ICU patients who were transferred out earlier than the time point, the laboratory indicators, AGI grade, the amount of calories and protein, organ support, and other items at the time point were analyzed according to the data on the day of transfer.

### Statistical analysis

All data were tested for normal distribution. The continuous data not conforming to the normal distribution were expressed as median (interquartile range, IQR). Nonparametric tests were used to compare the two groups. The categorical variables were described as n (%) and analyzed using the chi-square test or Fisher’s exact test. Univariable logistic regression was used for risk factor screening; variables with *P*-values < 0.05 were included in the multivariable logistic regression for further validation. The survival analysis was performed using the Kaplan–Meier method, and the curves were compared using the log-rank test. COX regression also used for survival. SPSS 22.0 (IBM Corp.) was used for statistical analysis in this study. Two-sided *P*-values < 0.05 were considered statistically significant. The Cochran–Armitage Trend Test was conducted to test the trend in categorical variables. Jonckheere–Terpstra test is used to determine whether two or more independent samples are derived from the same distribution. The Mantel–Haenszel Test is used to analyze the association between two categorical variables, taking into account the influence of one or more stratified variables.

### Ethical approval

The study was conducted in accordance with the Declaration of Helsinki and approved by the Ethics Committee of Ruijin Hospital of Shanghai Jiaotong University School of Medicine, China (Project identification code 2017-78, protocol code 1.0, and date of approval 2017-3-30). All participants provided written informed consent for participation in this study. I confirm that all methods were performed in accordance with the relevant guidelines. All procedures were performed in accordance with the ethical standards laid down in the 1964 Declaration of Helsinki and its later amendments.

## Results

### Baseline data of patients

**Figure 1:**
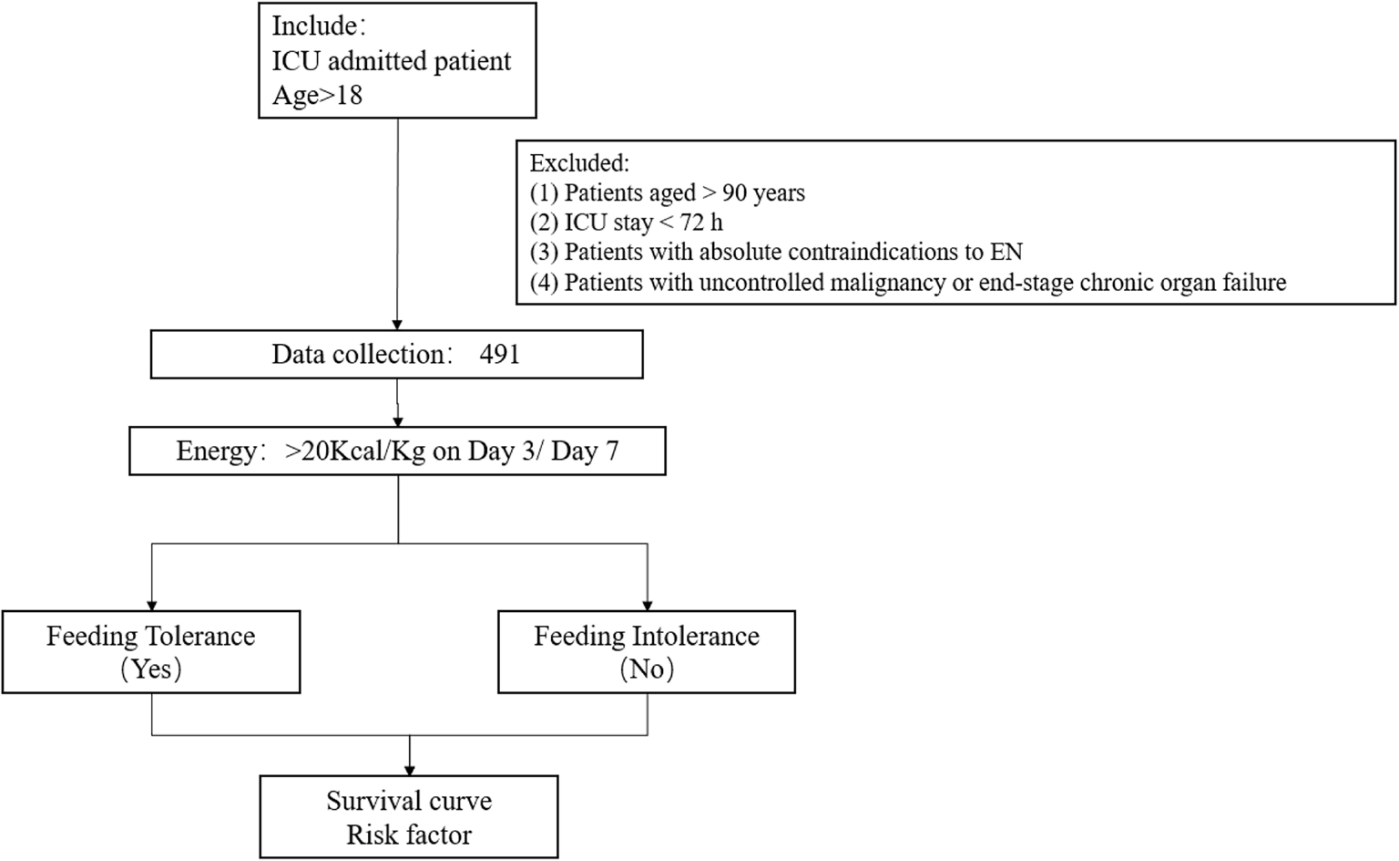
The flowchart about inclusion and exclusion.

A total of 15 ICUs in SHANGHAI participated in this study, and 491 patients were finally included in the analysis. The flowchart about inclusion and exclusion was in Fig. 1. The basic information of all the patients was shown in (Table 1).

**Table 1:** General characteristics of patients.

Characteristics	Value
Sex (n, %)	
Male	319 (64.97)
Female	172 (35.03)
Age (year), median (IQR)	66 (53, 77)
APACHE II on ICU admission, median (IQR)	14 (9, 19)
Height (cm), median (IQR)	168(160, 172)
Weight (kg), median (IQR)	65 (60, 70)
BMI (kg/m^2^), median (IQR)	23.03 (20.81, 24.73)
NRS2002 on admission to ICU (score), median (IQR)	4 (3, 5)
NRS2002 > 5score (cases, %), median (IQR)	88 (17.92%)
mNutric on admission to ICU (score)	3 (2, 5)
mNutric ≥ 5 scores (n, %)	92 (18.73)
Target calorie (kcal/day), median (IQR)	1500 (1300, 1750)
Target protein (g/day), median (IQR)	60 (50, 80)
Comorbidities (n, %)	326 (66.39)
≥ 2 types of comorbidities (n, %)	177 (36.05)
≥ 3 types of comorbidities (n, %)	85 (17.31)
Chronic kidney disease (n, %)	23 (4.68)
Chronic obstructive pulmonary disease (n, %)	40(8.13)
Hypertension (n, %)	193 (39.30)
Coronary heart disease (n, %)	76 (15.47)
Tumor (n, %)	62 (12.63)
Cerebrovascular accident (n, %)	79 (16.09)
Immune system disease (n, %)	8 (1.63)
Diabetes (n, %)	30 (6.11)
Others (n, %)	82 (16.70)
Main organ system involved (n, %)	
Respiratory system	262 (55.36)
Circulatory system	187 (38.09)
Urinary system	39 (7.94)
Coagulation system	35 (7.13)
Digestive system	134 (27.29)
Nervous system	131 (26.68)
Acid–base electrolyte imbalance	47 (9.57%)
Endocrine system	21 (4.27%)
Numbers of organ system involved(n, %)	
One system involved	241 (49.08%)
Two or more systems involved	250 (50.91%)
Three or more systems involved	88 (17.92%)
ICU stay time (day), median (IQR)	12.5 (7, 23)
ICU cost (10,000 RMB), median (IQR)	6 (3.21, 9.66)
28-day mortality rate (n, %)	61 (12.42)
SOFA (score), median (IQR)	3 (1, 5)
Total calorie intake (kcal/d), median (IQR)	1120 (800, 1500)
Total calorie intake (kcal/kg/d), median (IQR)	18.3 (12.1, 24)
Total protein intake (g/d), median (IQR)	50 (30, 70)
Total protein intake (g/kg/d), median (IQR)	18.3 (12.1, 24)
EN Calorie intake (kcal/day), median (IQR)	1000 (500, 1500)
EN calorie/total calorie (%), median (IQR)	98 (79, 10)
CRP (mg/L), median (IQR)	17.4 (4, 52)
PCT (ng/ml), median (IQR)	0.19 (0.05, 0.87)
p-ALB (mg/L), median (IQR)	97 (38, 183)
Total protein (g/L), median (IQR)	60 (54, 67)
ALB (g/L), median (IQR)	32 (29, 36)

### Nutrition implementation on days 1, 3, 7, and 14 of ICU stay

The data on the nutrition therapy on days 1, 3, 7, and 14 of ICU admission of the participants are shown in Table 2. The incidence of intolerance symptoms decreased on the 1st, 3rd, 7th, and 14th days of ICU admission (Table 2).

**Table 2:** Characteristics of EN treatment of ICU patients in SHANGHAI.

Variables	Day 1	Day 3	Day 7	Day 14	P^@^
SOFA score	5 (3,7)	4 (3,7)	6 (4,8)	4 (2,6)	0.210†
Prescriptions’ Total calorie (kcal/d)	500 (0,1000)	1000 (750,1300)	1000 (600,1500)	1100 (900,1500)	0.010*†
Prescriptions’ Total protein (g/d)	20 (0,40)	34 (8,40)	40 (20,60)	50 (35,60)	0.010*†
Total calorie intake (kcal/d)	462 (300,1020)	1000 (450,1250)	1000 (600,1400)	1150 (1000,1350)	< 0.001*†
kcal/kg/d	10.13 (7.67,16.67)	15 (9.00,18.00)	16.00 (10.00,20.00)	18 (16.67,20.36)	< 0.001†
Ratio to target calorie (%)	6.50 (0, 59.02)	58.00 (28.50,84.75)	69.67 (33.21,94.67)	88.56 (66.67,100.00)	0.010*†
Total protein intake (g/d)	18 (0,40)	36 (30,55)	40 (20,60)	60 (40,70)	0.010*†
Total protein intake (g/kg/d)	0.31 (0,0.62)	0.53 (0.42,0.82)	0.6 (0.28,0.80)	0.91 (0.67,1.23)	0.010*†
Ratio to target protein (%)	6.78 (0,65.20)	60.00 (23.67,81.33)	68.25 (34.34,97.67)	90.97 (60.71,110.00)	0.010*†
Patients of EN (n, %)	201 (40.94%)	432 (87.98%)	469 (95.52%)	485 (98.78%)	
Actual calorie of EN (kcal/d)	450 (0,900)	1000 (500,1100)	1000 (500,1100)	1100 (900,1500)	0.010*†
Actual protein of EN (g/d)	10 (0,40)	36 (20,50)	40 (20,60)	50 (35,60)	0.01*†
The ratio of EN calorie to target calorie (%)	7.00 (0,58.05)	58.05 (28.21,84.22)	66.67 (33.33,93.67)	77.00 (53.00,100)	0.030*†
The number of EN < 20 kcal/kg/d (EN intolercnce)	491 (100%)	407 (82.07%)	347(70.61%)	256 (52.23%)	0.020*†
The ratio of EN protein to target protein (%)	6.66 (0,62.21)	60.00 (23.33,82.00)	66.67 (34.34,92.67)	80.00 (52.00,100)	0.040*†
Patients of PN (n, %)	96 (19.55%)	214 (43.58%)	190 (38.70%)	105 (21.38%)	
PN calorie (kcal/d)	300 (200,545)	339 (200,150)	365 (190,865)	232 (164,640)	< 0.001*†
PN amino acid (g)	10 (0,40)	20.6 (10,40)	21 (10,40)	20.62 (12.05,42.50)	< 0.001*†
Laboratory indicators					
p-ALB (mg/L)	78.5 (38,160)	62.7 (35,132)	88.5 (38,168)	102 (40,176)	0.980†
CRP (mg/L)	55.6 (12.5,118)	50 (10.5, 107)	39 (12,94)	21.10 (6.60,62.33)	< 0.001*†
ALB (g/L)	31 (28,36)	31 (28,36)	32 (26,36)	32 (29,36)	1.000†
PCT (ng/ml)	0.34 (0.06,2.24)	0.25 (0.06,1.76)	0.19 (0.06,0.80)	0.14 (0.05,0.54)	< 0.001*†
Hypoglycemia (n, %)	20 (4.07)	13 (2.64)	12 (2.44)	6 (1.22)	0.030^&^*
AGI (n, %)					< 0.001*^#^
No AGI	18 (3.66)	75 (15.27)	260 (52.95)	326 (66.19)	< 0.001*^&^
AGI grade I	325 (66.19)	307 (62.53)	194 (39.51)	146 (29.74)	0.03*^&^
AGI grade II	81 (16.49)	71 (14.46)	29 (5.91)	14 (2.85)	0.04*^&^
AGI grade III	40 (8.14)	23 (4.68)	7 (1.43)	3 (0.61)	0.04*^&^
AGI grade IV	27 (5.49)	15 (3.05)	1 (0.20)	2 (0.40)	0.06*^&^
Physicians’ judgment on tolerance (n, %)					< 0.001*#
Intolerance	71 (14.46%)	49 (9.97%)	22 (4.48%)	7(1.42%)	< 0.001*^&^
Intermediate	177 (36.04%)	103 (20.97%)	17 (35.03%)	10 (2.04%)	0.05*^&^
Tolerance	243 (49.49%)	339 (69.04)	297 (60.49%)	474(96.54%)	0.04*^&^
The symptoms of intolerance (n, %)					< 0.001*#
Abdominal distension	375 (76.37%)	354 (72.10%)	160 (32.59%)	25 (5.10%)	< 0.001*^&^
Nausea/vomiting	311 (63.34%)	211 (42.62%)	108 (21.81%)	2(0.41%)	< 0.001*^&^
Abdominal pain	36 (7.33%)	37 (7.53%)	0 (0.00%)	0 (0.00%)	0.05*^&^
Diarrhea	2 (0.41%)	104 (21.18%)	65 (13.23%)	4 (0.81%)	0.07*^&^
Intestinal obstruction	0 (0.00%)	5 (1.02%)	1 (0.20%)	2 (0.41%)	0.09*^&^
Gastrointestinal bleeding	0 (0.00%)	3(0.61%)	1 (0.20%)	2 (0.41%)	0.21*^&^
Increased intra-abdominal pressure	1 (0.20%)	4 (0.81%)	1 (0.20%)	1 (0.20%)	0.25*^&^
No symptoms (n,%)	23 (4.68%)	104 (21.18%)	276 (56.10%)	466 (94.90%)	< 0.001*^&^

@: All comparisons are based on days 1, 3, and 7 data comparison; *P < 0.05;

#: Mantel–Haenszel test.

†: Jonckheere–Terpstra test.

&: Cochran–Armitage trend test.

*EN* Enteral nutrition, *Icus* Intensive care units.

### Comparison of EN implementation in ICU patients with different prognoses

Univariable regression analyses were carried out with 28-day mortality as the outcome. Age, admission mNutric score, ICU APACHE II score, ICU SOFA score, whether calorie intake reached 20 kcal/kg/day on days 3 and 7, whether protein intake reached 0.5 g/kg/day (the median total protein intake) on days 7 is associated with the outcome on day 28 (Table S1). Multivariable logistic regression was performed on the above parameters, and the results showed that age, mNutric score on admission, and protein intake < 0.5 g/kg/day on day 7 were independent risk factors for 28-day mortality. (Table S1).

### The effect of EN intake on the survival of ICU patients

**Figure 2:**
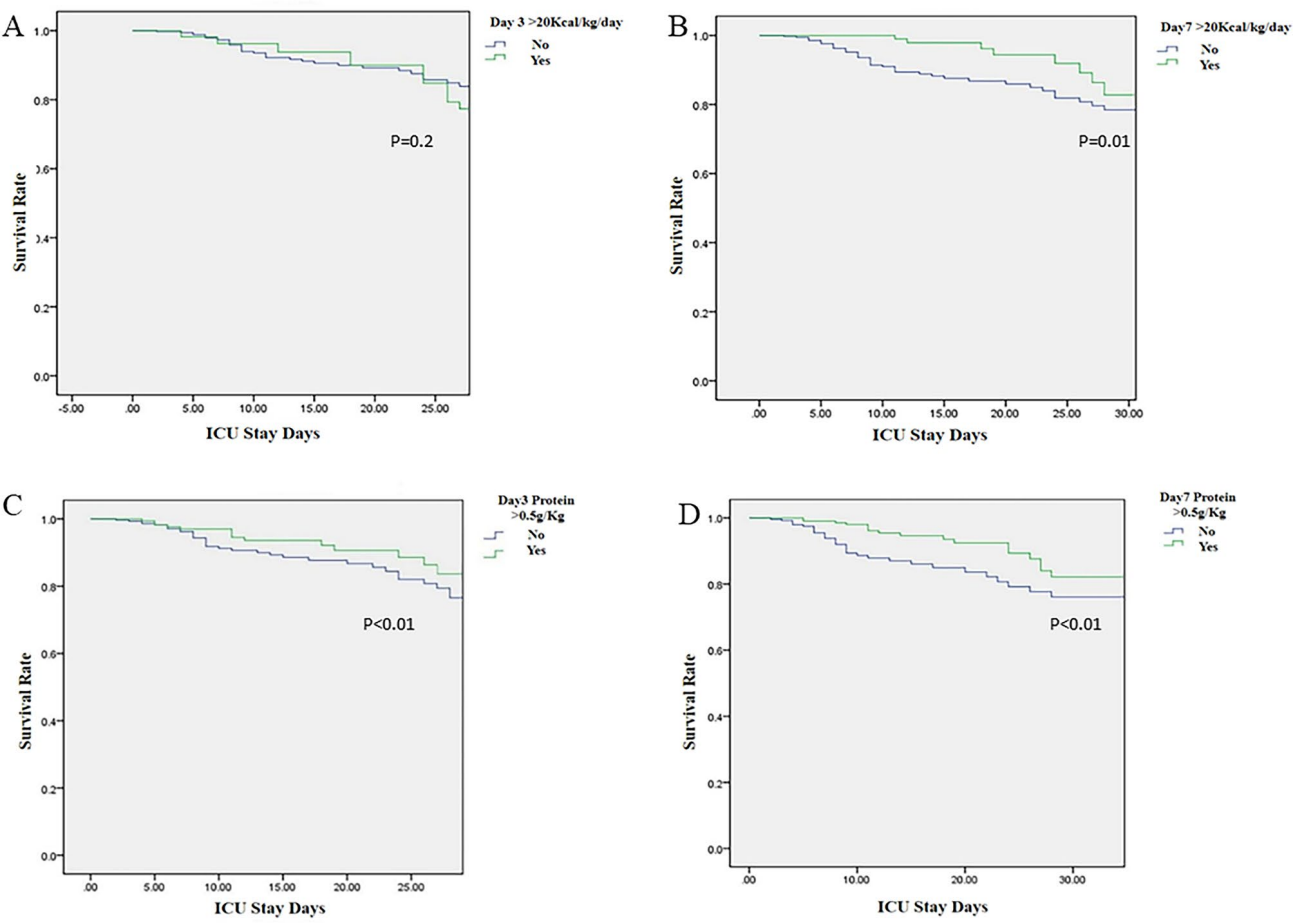
Kaplan–Meier survival analysis of the patient’s 28-day mortality on whether calories reached 20 kcal/kg/day (**A**) on day 3 (χ^2^ = 5.59, *P* = 0.2) or (**B**) on day 7 (χ^2^ = 13.09, *P* = 0.010); (**C**) whether the protein reached 0.5 g/kg/day on day 3 (χ^2^ = 13.99, *P* < 0.010) or (**D**) on day 7 (χ^2^ = 14.27, *P* < 0.010).

Taking the 28-day survival status as the endpoint, the comparison between EN reached 20 kcal/kg/day on day 3 was listed (Table S2). The survival rate was no significant difference. However, Kaplan–Meier survival analyses were performed on whether calories reached 20 kcal/kg/day on day 3 (Fig. 2A or day 7 (Fig. 2B) of ICU admission. The results showed that the survival curve of patients with EN tolerance on day 7 of ICU admission was better than in patients with intolerance (*P* = 0.01), while there were no differences on day 3 (*P* = 0.2). The Cox regression shows the same patten: Day 3 *p* = 0.09, HR 0.83 95% CI (0.67,1.03), Day 7 *p* = 0.04, HR 0.82 95% CI (0.75,0.98). We adjusted age and ApacheII score in COX regression model.

### The effect of protein intake on the survival of ICU patients

Using the 28-day survival status as the endpoint, the comparison between whether the protein reached 0.5 g/kg/day on day 3 on day 3 or not was listed (Table S2). The survival rate was no significant difference. Kaplan–Meier survival analyses were performed on whether the protein reached 0.5 g/kg/day on day 3 (Fig. 2C) and day 7 (Fig. 2D) of ICU admission. The results showed that the survival curve of patients whose protein intake reached 0.5 g/kg/day on days 3 and 7 was better than those who did not reach 0.5 g/kg/day (Day3 *P* < 0.01, Day 7 *P* < 0.01). The Cox regression shows the same patten: Day3 *p* = 0.04, HR 0.80 95% CI (0.65, 1.00); Day 7 *p* < 0.01, HR 0.75 95% CI (0.65, 0.92). We adjusted age and ApacheII score in COX regression model.

### Influencing factors of EN intake in ICU patients on day 7

The factors that might influence EN tolerance were included in a logistic regression to analyze EN tolerance in ICU patients on day 7. Preliminary analysis by univariable logistic regression showed that the AGI grade on day 1 (OR = 0.53), the use of organ support on day 3 (OR = 1.80), and the subjective judgment of the physician on EN tolerance on day 3 (OR = 2.96) were statistically significant. We take those factors above (they are with statistically significant in univariable regression) to making multivariable logistic regression, it was found that the AGI grade on day 1 (OR = 0.46) and the use of any organ support on day 3 (OR = 2.08) had a significant impact on EN tolerance in critically ill patients on day 7 (Table 3).

**Table 3:** Logistic regression on the relationship between patients’ characteristics and EN tolerance (> 20 kcal/kg/day) on Day 7.

Variables	OR (95% CI)	*P*
Univariable logistic regression		
Age	1.00 (0.99, 1.01)	0.910
APACHE II score on ICU admission	1.02 (0.98, 0.91)	0.080
SOFA score on ICU admission	1.02 (0.95, 1.08)	0.670
mNutric score on ICU admission	1.03 (0.91, 1.16)	0.500
NRS2002 on ICU admission	1.07 (0.97, 1.18)	0.210
Number of fundamental diseases	1.00 (0.86, 1.6)	0.940
Number of organs involved upon ICU admission	0.79 (0.59, 1.04)	0.540
Post-pyloric feeding or not	0.63 (0.35, 1.15)	0.130
AGI grade on day 1	0.51 (0.42, 0.74)	< 0.001*
Any organ support on day 3	1.80 (1.21, 2.67)	0.030*
AGI grade on day 3	1.12 (0.89, 1.39)	0.330
The subjective judgment of the physician on EN tolerance on day 3	2.96 (1.37, 5.28)	< 0.001*
Multivariable Logistic regression		
AGI grade on day 1	0.41 (0.30, 0.62)	0.010*
Any organ support on day3	2.08 (1.29,3.34)	< 0.001*

*P < 0.05, all those factors with *p* < 0.05 in univariable regression was adjusted in multivariable logistic regression.

*EN* Enteral nutrition.

## Discussion

In this study, we collected clinical information in critically ill patients admitted to the ICUs in 15 hospitals in SHANGHAI. Our data suggest that enteral nutrition tolerance and adequate protein intake are important for improving survival in ICU patients, as we hypothesized.

### Nutritional assessment

Nutritional status and risk of malnutrition should be assessed first before nutritional treatment. Heyland^7^ And Jie^8^ conducted prospective non-randomized studies that showed that patients with high malnutrition risk were more likely to benefit from early EN. The NRS2002 and Nutric assessments are the most recommended assessment methods. It is necessary to emphasize that there are still controversies regarding the best tool for assessing the malnutrition risk^6^.Therefore, further research is needed to find more reasonable ways to evaluate the nutritional status of critical patients.

### Use of PN

In this study, 59.47% of the patients started PN at ICU admission, while 43.58% on day 3 and 38.69% on day 7. These results agree with Xing et al.^9^, suggesting that PN was started earlier in ICU patients in China. It is currently believed that PN should be the rescue remedy for EN. Nevertheless, compared with EN, the implementation and monitoring of PN is relatively simple, and it is easy to achieve the nutritional goals, which fits the situation of insufficient ICU resources in China^10^. The NUTRIREA-2 study showed^11^ that patients with full PN developed significantly fewer gastrointestinal symptoms, and PN did not increase the mortality rate and incidence of nosocomial infection.

### Calories and protein target setting and EN tolerance

Indirect calorimetry (IC) is considered the gold standard for calorie target setting in the ICU so as to prevent underfeeding and overfeeding^12–14^. In this study, the target calorie and protein were mainly calculated according to the guidelines, accounting for 56.82% of the cases. This is reasonable to use a metabolic formula since none of the centers in this study is equipped with IC. The recommendations for calculating target energy differ between guidelines^15, 16^. According to the ASPEN recommendation, the target calorie in our population was estimated to be 1350–1650 kcal/kg/day (the median weight was 65 kg), similar to the target calorie set by clinicians in the actual practice.

In this study, patients reaching 20 kcal/kg/day on day 3 did not show benefit on survival. Several studies^12, 17–19^ reported a higher need for organ support in full-energy supply patients than in underfed patients. The time of reaching the target calorie is controversial. It is believed that endogenous energy is produced in the acute stage, and nutrition support may lead to harmful overfeeding to patients^20^. An observational study found that meeting 70–80% of the target calorie within 1 week might be ideal^21^. Notably, the EN formulation used in this study was mostly 1 or 0.9 kcal/ml. Meeting the target energy intake requires about 1300 ml of EN, which increases the difficulty of fluid management in critically ill patients and might be unfavorable for the removal of organ support such as mechanical ventilation or renal replacement therapy. Meanwhile, on day 7, EN intake < 20 kcal/kg/day (intolerance) was happened in 70% patients, with the negative impact on 28-day survive (Kaplan–Meier survival analysis). This result is concordance with other reports^10, 11, 15, 16^, which reference the benefit to reaching EN tolerance within 7 days.

It is widely accepted that the protein intake in critically ill patients should be > 1 g/kg/day and at least 0.8 g/kg/day for general hospitalized patients^22, 23^. In this study, only about 50% of patients reached an intake of > 0.5 g/kg/day. In the NEED study by Ke et al.^24^, the average protein intake was 0.67 g/kg/day. In this study, logistic regression and the Kaplan–Meier survival analysis indicated that a protein intake < 0.5 g/kg/day on day 3 or 7 significantly affected 28-day mortality. These results are supported by other observational studies^25, 26^, emphasizing the importance of protein intake in the early stage of critical illness. Obviously, improving EN tolerance is helpful to reach the protein target.

### Risk factors of EN intolerance

An international nutrition survey showed that the average calorie intake in critically ill patients on day 7 was at 16.5 kcal/kg/day^27^. The 2017 Nutrition Day survey also showed that less than 25% of the patients reached a target volume of 25 kcal/kg/day within 2 weeks in the ICU^28^. According to the definition of EN intolerance, it is sure that most patients could be diagnosed with EN intolerance.

FI can also be diagnosed based on symptoms such as abdominal distension, nausea and vomiting, and gastric residual volume (GRV). Taking routine GRV measures is against the mainstream guidelines, while other symptoms lack an objective way of quantification^15, 16^. Using the actual feeding amount as the criterion for EN intolerance is more relative to the EN intolerance definition, and the method is objective and easy to standardize. Therefore, we defined EN intolerance according to whether the EN could reach 20 kcal/kg/day.

In this study, the main factors predicting EN tolerance on day 7 were AGI grade on day 1 and any organ support on day 3. A study showed that the AGI grade on day 1 was associated with future GI function and ICU outcomes^29^. The intestinal function of critically ill patients can often be improved after appropriate treatment and organ support (mechanical ventilation, renal replacement therapy, vasopressor, etc.). Thus, organ support means a higher probability of controlling the critical state, so there might be a higher possibility that the GI function could be preserved.

The predictors of EN tolerance are less studied because of the significant differences in the definition of EN tolerance, especially when tolerance is judged according to the symptoms^4^. Hu et al.^4^ reported that 15 factors, including pneumonia, nutritional preparation, shock, skin infection, continuous feeding, etc., were associated with tolerance and established a predictive model. Unfortunately, the number of patients was small, and the results need to be confirmed^4^.

In this study, 15.01% (n = 94) of patients received post-pyloric feeding in the ICU, which is a low level. Post-pyloric feeding can improve EN tolerance and thus improve nutritional intake^24^, but the regression analysis in this study showed that the use of post-pyloric feeding did not affect whether the patient’s calorie intake reached 20 kcal/kg/day.

### Limitations

This study has limitations. First, as an observational study, the causal relationship between EN treatment and the patient outcome cannot be established. Secondly, the number of hospitals involved in this study was less than half of the number of tertiary hospitals in SHANGHAI and did not cover the secondary hospitals, thus biasing the results. In addition, although this study included consecutive patients admitted over 1 month in 15 ICUs, the sample size was relatively small. Third, the study time in each center was about 1 month, and no further long-term prognosis of the patients was followed up. The impact of nutritional therapy on patients might be difficult to reflect on in the short term, and follow-up for long-term outcomes is necessary.

The institutions that participated in the study covered half of the city districts (8/16 districts in SHANGHAI) and were mainly tertiary hospitals that admitted patients from all over SHANGHAI. Thus, we believe the patients’ data are representative of the SHANGHAI ICU patients.

## Conclusions

The proportion of EN intolerance in ICU patients is high. Patients with better EN tolerance have a better 28-day survival rate. A protein intake on day 7 of < 0.5 g/kg/day is an important factor affecting patient survival at 28-day survival. Improving EN tolerance and protein intake in early ICU days may improve the outcomes of the patients.

### Supplementary Information

Supplementary Table S1.

Supplementary Table S2.

## Data Availability

All data generated or analysed during this study are included in this published article.
