# Supplementary material for: Improving enteral nutrition tolerance and protein intake maybe beneficial to intensive care unit patients
Source: Sci Rep. 2023 Dec 7;13:21614. doi: 10.1038/s41598-023-49050-z (PMC10703788; doi:10.1038/s41598-023-49050-z)
Supplement: Supplementary file 1 — Supplementary Table S1. [file 41598_2023_49050_MOESM1_ESM.docx]

**Table S1** Logistic regression on the relationship between nutritional treatment characteristics and 28-day mortality.

| Variables | OR (95%CI) | P |
| --- | --- | --- |
| Univariable regression analysis |  |  |
| Age | 0.97 (0.96, 0.97) | 0.040* |
| APACHE II score | 0.90 (0.88, 0.91) | 0.010* |
| SOFA score >2 points | 0.75 (0.71, 0.79) | 0.020* |
| mNutric score | 1.55 (1.15, 2.06) | 0.030* |
| EN calorie on Day3 >20Kcal/kg/d | 0.12 (0.05, 0.21) | 0.030* |
| EN protein on Day 3 reached 0.5 g/kg/d | 0.13 (0.08, 0.21) | 0.020* |
| Physicians’ subjective judgment on tolerance | 0.79 (0.59, 1.04) | 0.540 |
| EN calorie on day 7 >20 kcal/kg/d | 0.10 (0.05, 0.17) | 0.010* |
| EN protein on day 7 reached 0.5 g/kg/d | 0.78 (0.59, 0.94) | 0.040* |
| Multivariable logistic regression |  |  |
| Age | 0.97 (0.96, 0.99) | <0.001* |
| mNutric score | 1.28 (1.08, 1.50) | 0.040* |
| EN protein on day 7 reached 0.5 g/kg/d | 0.40 (0.22, 0.70) | 0.020* |

*: P <0.05, all those factors with p<0.05 in univariable regression was adjusted in multivariable logistic regression
