# Supplementary material for: Improving enteral nutrition tolerance and protein intake maybe beneficial to intensive care unit patients
Source: Sci Rep. 2023 Dec 7;13:21614. doi: 10.1038/s41598-023-49050-z (PMC10703788; doi:10.1038/s41598-023-49050-z)
Supplement: Supplementary file 2 — Supplementary Table S2. [file 41598_2023_49050_MOESM2_ESM.docx]

| Table S2.1: Comparison of enteral nutrition and clinical prognosis between EN tolerant group and intolerant group on Day 3 | | | |
| --- | --- | --- | --- |
| EN calorie > 20Kcal/kg/d | | | |
|  | **Tolerant  (n=88)** | **Intolerant (n=403)** | **P** |
| Age(year) | **66 (49,74)** | **65 (52,77)** | **0.24** |
| APACHE II on ICU admission (score) | **15 (10,18)** | **13 (9,18)** | **0.14** |
| Day3 calorie intake (Kcal/d) | **1300 (1000,1500)** | **180 (100,780)** | **0.01*** |
| Day3 protein intake (g/d) | **60 (42.5,65)** | **20 (0,40)** | **0.00*** |
| Day3 PN calorie intake (Kcal/d) | **0 (0,204)** | **0 (0,128)** | **0.3** |
| ICU stay time (d) | **15 (10,26)** | **13 (7,21)** | **0.06** |
| 28-day mortality (cases, %) | **9 (10.27)** | **52 (12.90)** | **0.31** |
| ICU mortality (cases, %) | **8(9.10)** | **55(13.64)** | **0.12** |
| Total protein intake>0.5 g/kg/d | | | |
|  | **>0.5g/kg/d**  **(n=193)** | **<0.5g/kg/d**  **(n=298)** | **P** |
| Day3 protein intake (g/d) | **42.5 (40,60)** | **7 (0,20)** | **0.00*** |
| Day3 PN amino acid (g/d) | **0 (0,10)** | **0 (0,31)** | **0.01*** |
| Day3 total calorie intake (Kcal/d) | **1000**  **(1000,1350)** | **0  (0,500)** | **0.00*** |
| ICU stay time (day) | **14 (9.75,25)** | **11 (6,21.5)** | **0.07** |
| 28-day mortality (cases, %) | **22 (11.39)** | **39 (13.08)** | **0.13** |
| ICU mortality (cases, %) | **26 (13.47)** | **37 (12.41)** | **0.93** |
| *：P<0.05 |  |  |  |

| **TableS2.2: Comparison of enteral nutrition and clinical prognosis between EN tolerant group and intolerant group on Day 7** | | | |
| --- | --- | --- | --- |
| **EN calorie > 20Kcal/kg/d** | | | |
|  | **Tolerant**  **(n=144)** | **Intolerant (n=347)** | **P** |
| Age(year) | **66 (53, 76)** | **65 (52, 78)** | **0.32** |
| APACHE II on ICU admission (score) | **15 (12,19)** | **13 (8, 18)** | **0.07** |
| Day3 calorie intake (Kcal/d) | **1300 (1000, 1500)** | **230 (100, 810)** | **0.03*** |
| Day3 protein intake (g/d) | **0 (0, 100)** | **0 (0, 365)** | **0.06** |
| Day7 PN calorie intake (Kcal/d) | **60 (75, 50)** | **25**  **（15, 40）** | **0.00*** |
| ICU stay time (d) | **15 (10, 26)** | **12 (6, 21)** | **0.06** |
| 28-day mortality (cases, %) | **12 (8.33)** | **49 (14.12)** | **0.07** |
| ICU mortality (cases, %) | **15 (10.41)** | **48 (13.83)** | **0.10** |
| **Total protein intake>0.5 g/kg/d** | | | |
|  | **>0.5g/kg/d (n=225)** | **<0.5g/kg/d**  **(n=266)** | **P** |
| Day7 protein intake (g/d) | **51 (40, 60)** | **18.38 (0, 20)** | **0.00*** |
| Day7 PN amino acid (g/d) | **0 (0, 10)** | **0 (0, 10)** | **0.33** |
| Day7 total calorie intake (Kcal/d) | **1220 (1000, 1500)** | **500 (100, 520)** | **0.01*** |
| ICU stay time (day) | **14 (10,25)** | **9 (6, 18.5)** | **0.08** |
| 28-day mortality (cases, %) | **23 (10.22)** | **38 (14.28)** | **0.09** |
| ICU mortality (cases, %) | **26 (11.56)** | **37 (13.90)** | **0.97** |
| *：P<0.05 | | | |
